# Metabolomic investigation of urinary extracellular vesicles for early detection and screening of lung cancer

**DOI:** 10.1186/s12951-023-01908-0

**Published:** 2023-05-16

**Authors:** Qinsi Yang, Jiaxin Luo, Hao Xu, Liu Huang, Xinxi Zhu, Hengrui Li, Rui Yang, Bo Peng, Da Sun, Qingfu Zhu, Fei Liu

**Affiliations:** 1 Wenzhou Institute, University of Chinese Academy of Sciences, Wenzhou, Zhejiang 325000 China; 2 National Engineering Research Center of Ophthalmology and Optometry, Eye Hospital, Wenzhou Medical University, Wenzhou, 325027 China; 3 Department of Oncology, Tongji Hospital, Tongji Medical College, Huazhong University of Science and Technology, Wuhan, 430030 Hubei China; 4 Key Laboratory of Heart and Lung, The First Affiliated Hospital of Wenzhou Medical University, Wenzhou, 325000 Zhejiang China; 5 Institute of Life Sciences & Engineering Laboratory of Zhejiang Province for Pharmaceutical Development of Growth Factors, Wenzhou University, Wenzhou, 325035 China; 6 The First Affiliated Hospital of Wenzhou Medical University, Wenzhou, 325035 Zhejiang China

**Keywords:** Lung cancer, Metabolomics, Extracellular vesicles, Early diagnosis

## Abstract

Lung cancer is a prevalent cancer type worldwide that often remains asymptomatic in its early stages and is frequently diagnosed at an advanced stage with a poor prognosis due to the lack of effective diagnostic techniques and molecular biomarkers. However, emerging evidence suggests that extracellular vesicles (EVs) may promote lung cancer cell proliferation and metastasis, and modulate the anti-tumor immune response in lung cancer carcinogenesis, making them potential biomarkers for early cancer detection. To investigate the potential of urinary EVs for non-invasive detection and screening of patients at early stages, we studied metabolomic signatures of lung cancer. Specifically, we conducted metabolomic analysis of 102 EV samples and identified metabolome profiles of urinary EVs, including organic acids and derivatives, lipids and lipid-like molecules, organheterocyclic compounds, and benzenoids. Using machine learning with a random forest model, we screened for potential markers of lung cancer and identified a marker panel consisting of Kanzonol Z, Xanthosine, Nervonyl carnitine, and 3,4-Dihydroxybenzaldehyde, which exhibited a diagnostic potency of 96% for the testing cohort (AUC value). Importantly, this marker panel also demonstrated effective prediction for the validation set, with an AUC value of 84%, indicating the reliability of the marker screening process. Our findings suggest that the metabolomic analysis of urinary EVs provides a promising source of non-invasive markers for lung cancer diagnostics. We believe that the EV metabolic signatures could be used to develop clinical applications for the early detection and screening of lung cancer, potentially improving patient outcomes.

**Supplementary Information:**

The online version contains supplementary material available at 10.1186/s12951-023-01908-0.

## Introduction

Lung cancer is the malignant tumor with the highest morbidity worldwide, and due to the lack of effective early diagnostic methods. Over 61% of lung cancer patients are diagnosed in advanced stages III and IV, with a bleak 5-year survival rate of only 4%. In contrast, early-stage cancer patients have a much more promising 5-year survival rate of approximately 50% [1, 2]. Therefore, finding a stable, repeatable, and non-invasive biomarker would be crucial for developing a screening method for early detection of lung cancer [2]. EVs are released by almost all living cells and can be isolated from various biofluids including urine. EVs, mainly including exosomes (30–150 nm) and microvesicles (100-1,000 nm), are membrane vesicles encapsulated with lipid bilayers bearing molecular markers of their parental tumor cells[3–5]. Numerous studies have identified tumor-related biomarkers in EVs, with their types and expression levels closely associated with the development of certain cancers[6–12]. Earlier studies focused on urogenital-related cancers and led to the identification of protein, RNA, lipid and metabolite biomarkers in prostate cancer [13–15], bladder cancer [16, 17] and kidney cancer [18, 19]. The advantages of urinary EVs include their non-invasiveness, high stability, and ease of processing. Importantly, urinary exosomes are found genetically related to multiple cells and tissues, and may be harnessed as a potential marker source for noninvasive liquid biopsy in cancer diagnostics[20]. A previous study screened non-small cell lung cancer (NSCLC)-associated proteins by comparing the urinary exosomal proteome of normal controls and NSCLC patients, suggesting that LRG1 may be a candidate biomarker for NSCLC diagnosis in urine[10]. Thus, urinary EVs isolates were able to detect disease-specific molecules undetectable in urine, either because of their low concentration in the bulk fluid or because of their location on EVs [21]. In fact, EVs could serve as a more specific source for biomarker discovery than unfractionated urine, and provide precision diagnostic information without invasive [22, 23]. Previous studies have shown that the biomolecules change at EVs level occurs earlier than that in body fluids [24]. Thus, EVs are expected to serve as biomarkers for disease diagnosis, target therapy, drug carriers, and prognostic analysis [25, 26].

There are certain distinctions in the genomes of cancer cells and tissues in different cancer patients. Due to the heterogeneity of cancer cells, the sensitivity and specificity of many genetic and protein diagnostic markers are greatly limited, which may be effective in identifying tumors in some patients but ineffective in others[27]. In contrast, cancer cell proliferation contains various metabolic processes, of which metabolomics could provide a quantitative and qualitative method to screen metabolic biomarkers (molecular weight < 1,000 Da) in biological samples[28]. EVs carry parent cell-derived bioactive substances and excrete small metabolic molecules and lipids into the circulatory system. These small molecules can participate in diverse physiological and pathological processes in a biological system. For example, lipids from EVs are thought to mediate extracellular communication, such as immune activation or inhibition, so they are highly related to many types of immune diseases[29].

Many studies have focused on the analysis of EV RNAs [30, 31] and proteins[32]. Metabolite changes occur downstream of gene and protein regulation, therefore are more likely to reveal dynamic changes in biological status. However, not much attentions have been paid to small molecule metabolites, especially the investigation of cancer biomarkers via urinary EVs. As autonomous metabolic reactors, EVs are capable of delivering specific and functional metabolites into the tumor microenvironment [33]. Additionally, metabolic lipids have a crucial function in exosome biogenesis and interact with the tumor microenvironment (TME) to influence tumorigenesis and progression[34]. With the increase of specific enrichment and normalization methods, EV metabolomics could be used to gain novel biomarkers[35, 36]. In this study, metabolomic analysis based on liquid chromatography-tandem mass spectrometry (LC-MS/MS) is performed to assess the metabolite profile of urinary EVs from different stages of lung cancer and to discover specific novel biomarkers for early detection and non-invasively screening of lung cancer. This study fills a gap in finding and validating urinary EV metabolites as diagnostic biomarkers for lung cancer in a larger cohort of patients.

## Materials and methods

### Clinical samples and EV isolation

All urine samples were obtained from the Tongji Hospital in the Tongji Medical College at Huazhong University of Science and Technology for research upon informed consent from corresponding ethics committee. Table 1 shows the information of lung cancer patients and healthy individuals. All patients with lung cancer were diagnosed by pathology, imaging, and cytology, which were staged as stage I, stage II, stage III, and stage IV according to the Union for International Cancer Control criteria. Stage I and stage II are classified as early lung cancer, and Stage III and stage IV are classified as advanced lung cancer. The criteria used to classify each stage include the size of the tumor, the extent of tumor spread to nearby lymph nodes, and the presence of metastasis. The control subjects were from people who came to the hospital for regular check-ups and were identified without tumors and urinary tract infections. Since urine values vary considerably during a 24-hour period, the midstream specimen of the first-morning urine was collected for all patients.The urine was collected in a 50-mL tube, and then centrifuged at 2,000 × g for 10 min, and the supernatant was immediately frozen at − 80 ºC. The frozen urine samples were thawed on ice and then filtered through a 0.22 μm filter (Sigma-Aldrich Chemie GmbH, Taufkirchen, Germany). The urine sample was then loaded into the EXODUS device[37] to acquire highly purified EVs.

**Table 1:** Clinical information of lung cancer patients

Disease status	Clinical stage	N	Age, years Median (range)	Gender N (male/female)	Current or former smoker N (Yes/No)	Pathological type N (Adenocarcinoma/ Squamous carcinoma/unknown)
Lung cancer	I	19 (18.6%)	60 (49–71)	10/9	6/13	16/3
	II	14 (13.7%)	59 (48–68)	12/2	10/4	10/4/0
	III	28 (27.5%)	56 (31–79)	24/4	14/14	13/12/3
	IV	14 (13.7%)	61 (47–77)	8/6	7/7	7/3/4
Normal control	/	27 (26.5%)	61 (51–77)	14/13	/	/

### Western blotting

The protein concentration of EVs was measured by a Qubit Kit (Thermo Fisher Scientific Inc., MA, USA) and the protein mixture was then separated by sodium dodecyl sulfate-polyacrylamide gel electrophoresis (SDS-PAGE). Briefly, the proteins were separated using a precast polyacrylamide mini-gels (Tri-glycine pH 8.3) with a Mini Trans-Blot module (Bio-Rad Laboratories Inc., CA, USA). Then, the proteins on gels were then electrically transferred onto polyvinylidene fluoride membrane, which were then blocked in PBST containing 5% fat-free milk powder. After that, the membranes were incubated with the primary antibody overnight at 4 ^o^C. The following antibodies were diluted by blocking liquid for western blot analysis, including anti-CD63, anti-CD9, anti-LRG1 (Abcam plc, Cambridge, UK), and anti-CD81 (Santa Cruz Biotechnology Inc., CA, USA). Thereafter, the membrane has been washed incubated with the secondary antibody (HRP-conjugated anti-mouse IgG or HRP-conjugated anti-rabbit IgG). Finally, the secondary antibody was washed by PBST 3 times, we used the enhanced chemiluminescence for immunodetection (PeiQing Science & Technology Co. Ltd., Shanghai, China) for imaging.

### Nanoparticle tracking analysis (NTA)

The Nanosight NS300 (Malvern Instruments Ltd., Malvern, UK) instrument was calibrated with the known concentrations of 100 nm pure standards to obtain optimum acquisition detector settings and post-acquisition settings. EV samples were diluted in PBS to obtain the ideal concentration (~ 20–60 particles per field of view) to achieve optimal counting. Perform NTA on the diluted samples according to the instructions provided by the manufacturer. Using the NanoSight NS300, each sample was recorded 3 times with a capture time of 30 s and analyzed with the camera level 15 and the detection threshold 5. Preferably, a syringe pump system (recommended infusion rate: 30 arbitrary units) is integrated into the setup to increase the statistical power of the measurement.

### Transmission electron microscope (TEM)

The EVs were fixed with 4% PFA and let stand for 30 min at room temperature. Use a pipette to place 20 µL of EV suspension on a clean Parafilm. In this case, the grids floated on the drop with their coated side facing the suspension for 30 min. The grids (membrane side down) were transferred to drops of PBS with clean forceps for 1 min. Fix the sample by incubating the grid with 1% glutaraldehyde for 5 min. Wash the grids eight times for 1 min each in 20 µL of distilled water. The sample was loaded on the grid and stained with 2% uranyl acetate for 30 s on ice. After air drying, imaging of the EVs was performed by FEI Talos F200S TEM (Thermo Fisher Scientific Inc., MA, USA).

### Metabolites extraction

The 2.0 ⋅ 10^9^ EV particles for each sample were applied for metabolomic analysis. The EV sample with a volume 100 µL was mixed with 300 µL of extract solution (acetonitrile: methanol = 1: 1, containing isotopically labeled internal standard mixture). The mixed samples were then vortexed and sonicated in an ice-water bath, which were left for 1 h at -40 ^o^C to precipitate proteins. After that, the sample was centrifuged at 12,000 rpm for 15 min, and the supernatant was collected and stored at -80 ^o^C until use. The quality control sample was prepared by mixing an equal aliquot of the supernatants from all of the samples.

### LC-MS/MS analysis

Liquid chromatography was performed using a UHPLC system (Vanquish, Thermo Fisher Scientific) with a UPLC BEH Amide column (2.1 mm × 100 mm, 1.7 μm), coupled to a Q Exactive HFX mass spectrometer (Orbitrap, Thermo Fisher Scientific Inc., MA, USA). The mobile phase A was 25 mmol/L ammonium acetate and 25 ammonia hydroxide in water (pH 9.75), and the mobile phase B was acetonitrile. The auto-sampler temperature was 4 ^o^C, and the sample injection volume was 4 µL. The QE HFX mass spectrometer was used to acquire MS/MS spectra with an information-dependent acquisition (IDA) mode using acquisition software (Xcalibur V2.2, Thermo Fisher Scientific Inc., MA, USA). In this mode, the acquisition software continuously evaluates the full scan MS spectrum. The ESI source conditions were set as follows: sheath gas flow rate as 25 Arb, Aux gas flow rate as 20 Arb, capillary temperature 350 ^o^C, full MS resolution as 60,000, MS/MS resolution as 7,500, collision energy as 10/30/60 in NCE mode, spray Voltage as 3.6 kV (positive) or -3.2 kV (negative), respectively.

### Bioinformatics analysis

We performed OPLS-DA to identify differences between two groups of data. The data was processed by SIMCA (V16.0.2, Sartorius Stedim Data Analytics AB, Umeå, Sweden), and then analyzed with an OPLS-DA model with 7-fold cross-validation. We used a volcano plot to show the differential metabolites between the two groups, and the selection criteria were a p-value < 0.05 (Student’s t-test) and VIP > 1. We calculated the Euclidean distance matrix for the quantitative values of differential metabolites and clustered the differential metabolites using a complete linkage method. The random forest model was created by randomly selecting a subset of features from the dataset and then building a decision tree model (random forest 4.6–14), which was trained on the dataset and used to make predictions.

### Data preprocessing and annotation

The raw data were converted to the mzXML format using ProteoWizard, which were then analyzed with an in-house program based on XCMS for peak detection, extraction, alignment, and integration. Then an in-house MS2 database (BiotreeDB, Shanghai Biotree Biotech Co. Ltd., Shanghai, China) was applied to metabolite annotation. The cutoff for annotation was set at 0.3. P < 0.01 was considered statistically significant.

## Results and discussions

### Clinical characteristics of subjects

To investigate metabolic signatures of lung cancer from urinary EVs, we collected urine samples from patients with Lung cancer (n = 75) and healthy control participants (n = 27). The Lung cancer patients were diagnosed at different stages according to the Union for International Cancer Control criteria: stage I (n = 19), stage II (n = 14), stage III (n = 28), and stage IV (n = 14). The detailed clinical information is shown in Table 1.

### Schematic workflow of this study via EV metabolomics

**Fig. 1:**
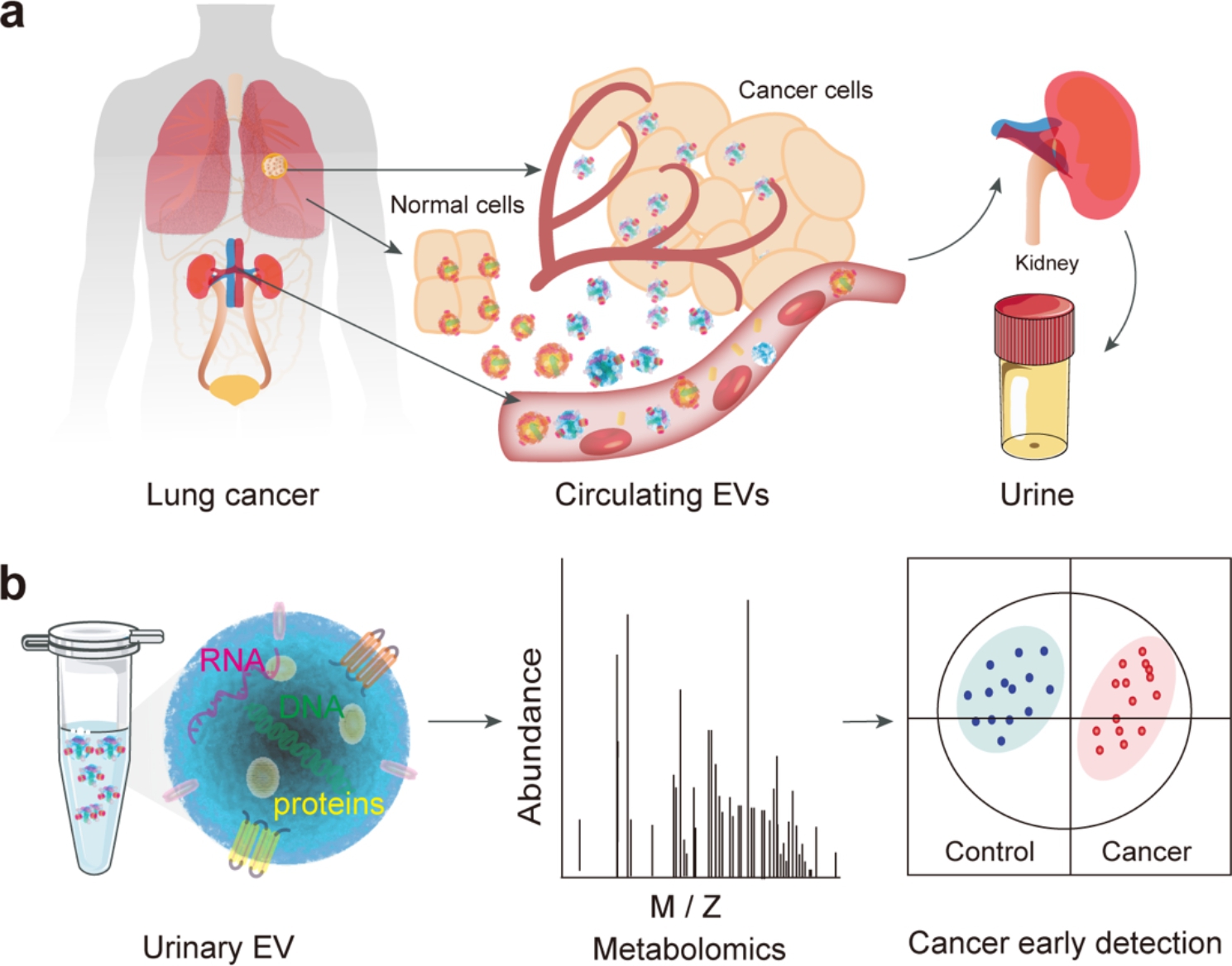
Illustration of workflow. (a) EVs are secreted by cells and released into the blood, and circulated in urine through hematuria exchange. (b) Urinay EVs are purified by EXODUS, followed by metabolomics and bioinformatics analysis for discovering potential biomarkers

The abscission of cancer cells is essentially a manifestation of increased migration and invasion of tumor cells. Since it has been shown that the differential expression of contents of EVs is closely related to lung cancer metastasis, which plays an important role in the multilink and multistep process (Fig. 1a). We chose urine-derived EVs as the study object for it is easily available and simpler composition than that blood. In the process of separation and purification, impurities such as protein fragments, lipids, and nucleic acids were filtered away through 20 nm diameter pores via the EXODUS device, while EV particles were left in the device. Then, the purified EV samples were transferred to microcentrifuge tubes and stored at -80 ^o^C (Fig. 1b). Subsequently, EV samples from normal individuals and lung cancer patients were subjected to metabolomic and bioinformatics analysis to discover differentially metabolized molecules that could navigate the early diagnosis of lung cancer patients and provide effective information for the development of lung cancer.

### Characterization of EVs

**Fig. 2:**
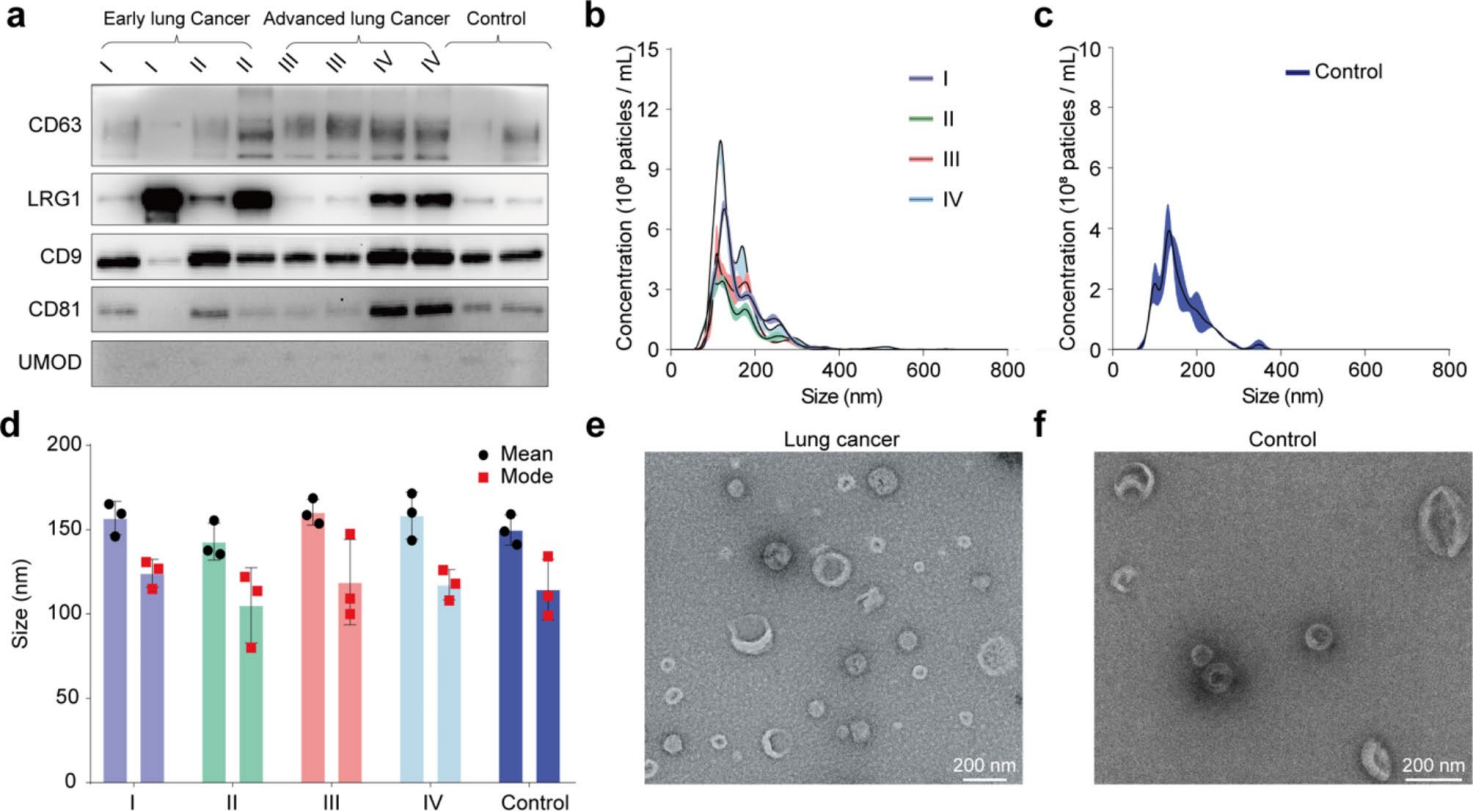
Characterization of EVs isolated from lung cancer patients and healthy doners. (a) Western blotting analysis of EV protein markers (CD63, CD9, CD81) and lung cancer-specific signature (LRG1) carried by urinary EVs. UMOD used as a negative control. An equal protein amount of 2 µg was loaded for all samples. (b) The distribution profiles of particle size from lung cancer at different stages, and healthy control in (c). (d) Characterizations of particle size from the lung cancer at different stages and control group, indicating that EVs are more abundant in patients with lung cancer. (e) Transmission Electron microscopy (TEM) analysis showing typica cup shape morphology of EVs from a lung cancer patient and (f) a healthy doner

The EVs were isolated from 5 mL urine depending on the EXODUS device. According to the guidelines of ISEV[38], Western blotting, NTA, and TEM were used to characterize urinary EVs (Fig. 2). The membrane proteins CD9, CD63, CD81 (common EV markers), and LRG 1 (Lung cancer-associated protein) were detected via western blotting. UMOD was used as a negative control, which showed no or very shallow bands. In general, the EV markers showed higher levels of lung cancer than that in healthy controls. Furthermore, lung cancer patients show a higher level of LRG 1 in EVs compared to healthy controls, indicating urinary EVs display close relations to lung cancer[12] (Fig. 2a). Additionally, the size distribution was compared by NTA analysis showing that EVs are more abundant in the patients’ group (Fig. 2b-c). The mean and mode size of EVs in the two groups are no significant difference (Fig. 2d). TEM images showed that the EVs ranged between 40 and 140 nm and have a cup-like morphology (Fig. 2d-f), and no significant difference can be seen regarding vesicle morphology beween the patient and helathy control. Since cancer cells exhibit enhanced production of EVs, the EV level is increased in the body fluids of cancer patients compared with healthy controls. Thus, EVs could perform as a rich source of non-invasive biomarkers for the diagnosis and prognosis of cancers, as well as therapeutic targets[39–41].

### Differential metabolic profiles of lung cancer and healthy control

We utilized high-resolution Q Exactive Orbitrap and quantitatively profiled the metabolites of urinary EVs derived from 75 lung cancer patients (33 early lung cancer and 42 advanced lung cancer) and 27 normal subjects to screen early diagnostic markers for lung cancer. Note that we applied particle number normalization in order to directly compare the metabolic differences in EVs between the control and lung cancer groups. The pathological status may significantly change the metabolic compositions of urinary EVs, and this composition variation might be accurately reflected when using the same number of EV particles for analysis. The metabolic difference between the control and lung cancer groups caused by variations in vesicle numbers will not be shown in this analysis.

**Fig. 3:**
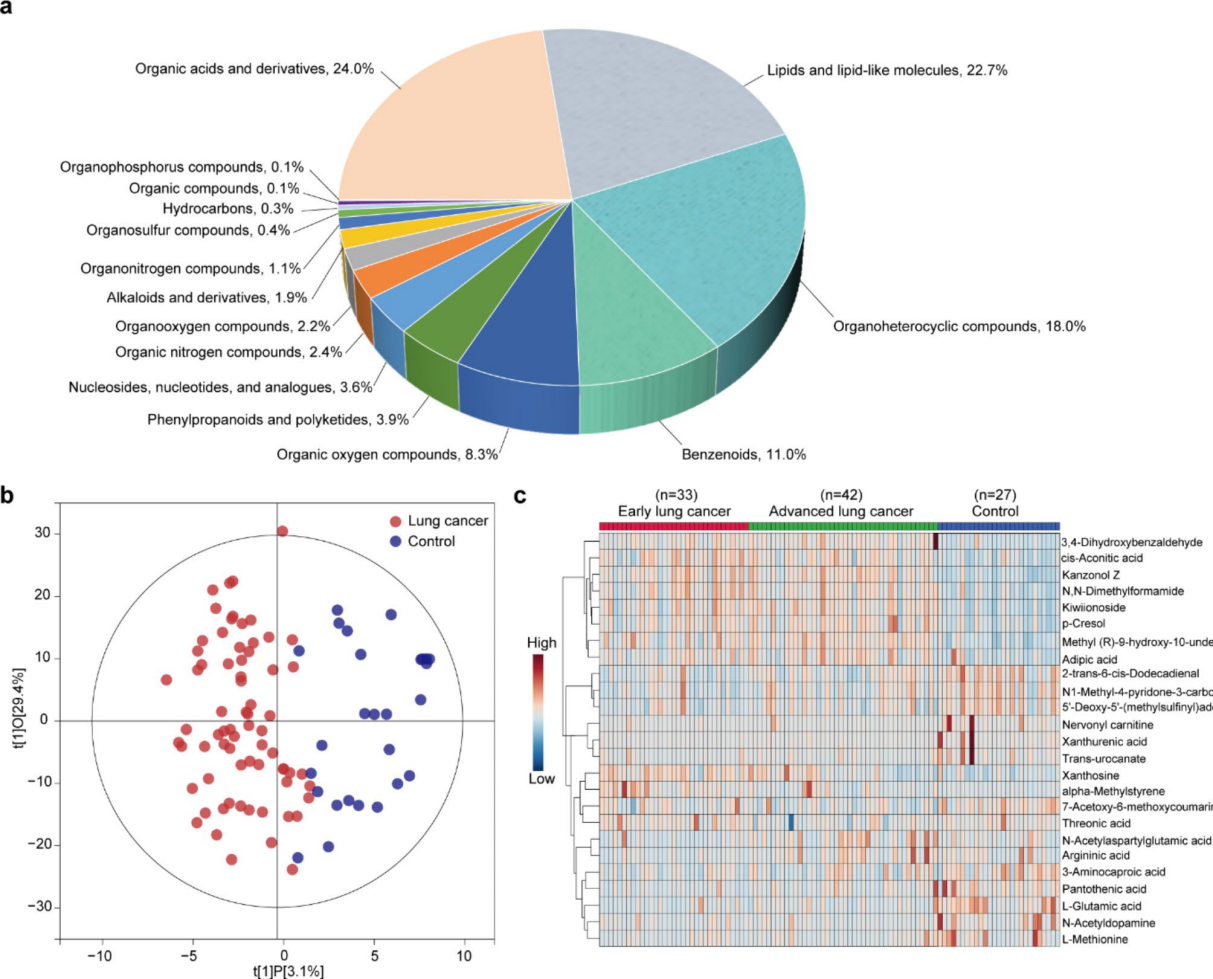
Analysis of metabolic profles of EVs from patients and controls. (a) Overall metabolic category of all identified metabolites. (b) Score scatter plot of OPLS-DA model for lung cancer and healthy control. Red dots represent the lung cancer group and blue dots represent healthy controls (c) Heat map showing the expression level of common metabolites in Control and lung cancer

A total of 698 metabolites were identified in metabolomics (Table S1). These detected metabolites were Organoheterocyclic compounds (18.0%), Organonitrogen compounds (1.1%), Organic acids and derivatives (24.0%), Organic compounds (0.1%), Nucleosides, nucleotides, and analogues (3.6%), Alkaloids and derivatives (1.9%), Organosulfur compounds (0.4%), Organooxygen compounds (2.2%), Phenylpropanoids and polyketides (3.9%), Organic oxygen compounds (8.3%), Organic nitrogen compounds (2.4%), Benzenoids (11.0%), Hydrocarbons (0.3%), Lipids and lipid-like molecules (22.7%), and Organophosphorus compounds (0.1%) (Fig. 3a). Among all metabolite categories, organic acid and its derivatives are the most abundant metabolite types accounting for 24%. Orthogonal Partial Least Squares Discriminant Analysis (OPLS-DA) was performed to give a snapshot of the metabolite characteristics of lung cancer and healthy control samples. The application of OPLS-DA aims to establish the relationship model between the metabolite expression and the sample category, to achieve the prediction of lung cancer. OPLS-DA t[1]P (abscissa) shows the predicted principal component from the first principal component, indicating the difference between sample groups; while the t[1]O (ordinate) shows the orthogonal principal component, indicating the difference within groups. Each scatter Dots represent a sample, in which red dots represent the lung cancer group and blue dots represent healthy controls. The OPLS-DA scores plot is based on the 698 metabolites (Fig. 3b) and the samples of lung cancer were clustered well away from the healthy control, indicating that the metabolic composition of lung cancer patients was remarkably different from those of healthy control. The most significant differential metabolites were shown in the heat map (Fig. 3c). We found 105 differential metabolites (Variable importance in projection (VIP) > 1 and Fold change (FC)> 1.2) including 83 up-regulated metabolites and 22 down-regulated metabolites of early lung cancer patients (Table S2). The KEGG enrichment analysis based on differential metabolites was engaged in Lysine degradation, Nicotine addiction, Neuroactive ligand − receptor interaction, Purine metabolism, Pyrimidine metabolism, Taurine and hypotaurine metabolism, D-Amino acid metabolism, Nicotinate and nicotinamide metabolism, ABC transporters, Steroid hormone biosynthesis, Metabolic pathways, Protein digestion and absorption, Glyoxylate and dicarboxylate metabolism, Glycine, serine and threonine metabolism, Arginine and proline metabolism (Figure S1a). The differential metabolites expression signature of urinary EVs holds great potential for the diagnosis of lung cancer. We subsequently assessed the ability of differential metabolites to distinguish lung cancer patients from healthy individuals by receiver operating characteristic (ROC) curve analysis. The area under curve (AUC) values corresponding to individual metabolites were listed (Table S3).

### Detection of lung cancer at early stages via EV metabolic signatures

**Fig. 4:**
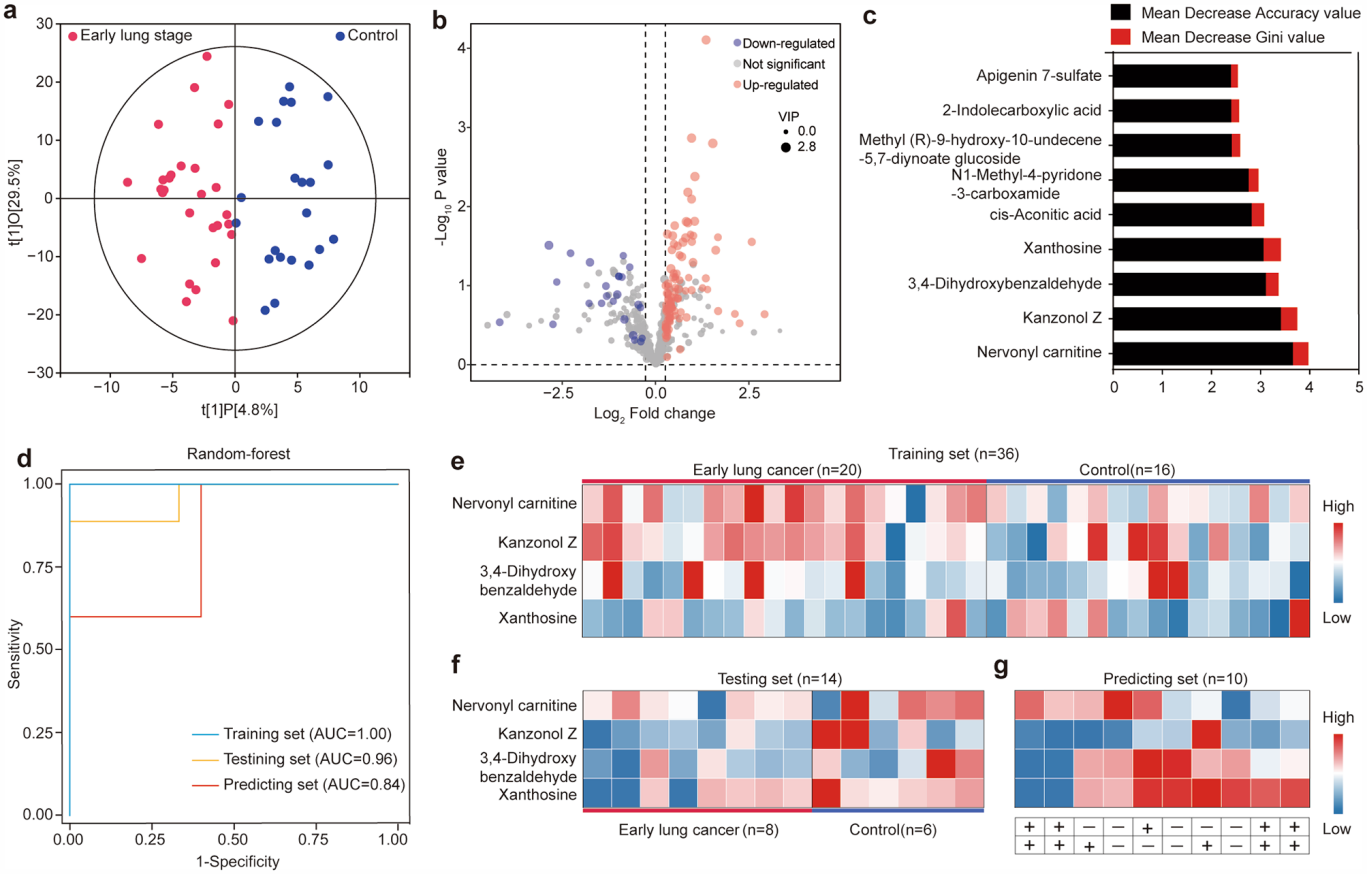
Investigation of metabolic markers for lung cancer detection and screening. (a) The OPLS-DA score plot model for differentiation of early lung cancer and healthy control. (b) Volcano plot showing expression levels of differential metabolites. (c) The top ten metabolites with the largest mean decrease accuracy value and mean decrease Gini value in the random-forest model. (d) The ROC curve was derived from the training set, testing set, and predicting set using four metabolites selected by the random-forest model, respectively. (e-g) Heat map showing the expression level of four metabolic markers in early lung cancer and control samples, including training set, testing set, and predicting set. “+” represents lung cancer patient, and “-” represents control.

Urinary-derived EVs metabolites were not only capable of identifying lung cancer but also showed great potential in the identification of early-stage lung cancer. According to the results of the OPLS-DA score plot, the two groups (28 early lung cancer and 22 healthy controls) were significantly distinguished within a high confidence interval (Hotelling’s T-squared ellipse) (Fig. 4a). We then performed differential analysis and discovered 125 differential metabolites (VIP > 1 and FC > 1.2) for the patients with early lung cancer compared with healthy controls (Table S4), including 101 up-regulated metabolites and 24 down-regulated metabolites. The expression levels of differential metabolites were shown in the volcano plot (Blue and red dots representing the down- and up-regulated differential metabolites, respectively) (Fig. 4b). The differential abundance (DA) analysis showed that the differential metabolites were involved in the KEGG pathway including Purine metabolism, beta-Alanine metabolism, Nicotinate, and nicotinamide metabolism, Pantothenate and CoA biosynthesis, ABC transporters, Steroid hormone biosynthesis, Carbon metabolism, Metabolic pathways, Bile secretion, Protein digestion and absorption, Butanoate metabolism, Glyoxylate, and dicarboxylate metabolism, Central carbon metabolism in cancer, Glycine, serine and threonine metabolism, Tyrosine metabolism (Figure S1b). Subsequently, we established a random forest model to discover early lung cancer based on all differential metabolites. According to the principle that the larger value of mean decrease accuracy and mean decrease Gini have the greater contribution to the random forest mode, a metabolic panel was selected composed of four metabolites with the largest contribution (Fig. 4c). AUC of the combination of Kanzonol Z, Xanthosine, Nervonyl carnitine, and 3,4-Dihydroxybenzaldehyde was up to 1 and 0.96 in the training set and testing set, respectively (Fig. 4d). The AUC of individual metabolites is shown in Figure S2. Xanthosine, the initial precursor of purine alkaloid synthesis, could also be used to differentiate normal sperm males from fertile individuals, which is a potential biomarker to assess normal sperm infertility[42]. In addition, Xanthosine can distinguish childhood asthma subtypes, and can be conducive to a deeper understanding of the underlying mechanisms of childhood asthma [43]. Nervonyl carnitine is one of the indicative metabolites for Aflatoxin B1 exposure. Studies have shown that the neuroprotective and anti-inflammatory effects of 3,4-Dihydroxybenzaldehyde are associated with selective modulation of microglial polarization and reduced production of inflammatory mediators and cytokines by inhibiting MAPK and NF-κB activation[44]. Thus, 3,4-Dihydroxybenzaldehyde might be a potential treatment for ischemic stroke and other neuroinflammatory diseases[45].

Expression levels of the selected metabolic signatures between lung cancer patients with early stages and healthy individuals are shown in Fig. 4e-f for the training set (n = 36) and testing set (n = 14), respectively, showing a clear difference between the two groups. Additionally, a validation set (n = 10) was included, and the AUC was achieved at 0.84 for the prediction of lung cancer at its early stages (Fig. 4d). The expression levels of signature metabolites are shown in Fig. 4g, in which 7 samples were correctly predicted out of 10 samples, including 4 lung cancer patients and 3 controls, indicating a good prediction potency.

## Conclusion

Precision diagnosis of lung cancer in its early stage is vital to improve treatment outcomes and increase patient survival rates. In this work, we present a non-invasive method based on metabolites carried by urinary EVs for early detection of lung cancer with high accuracy and specificity. We have systematically compared the metabolomic profiles of urinary EVs from lung cancer patients and healthy controls and identified a diagnostic panel composed of Kanzonol Z, Xanthosine, Nervonyl carnitine, and 3,4-Dihydroxybenzaldehyde. This diagnostic panel has been applied to the training set, testing set, and validation set, which can distinguish and predict lung cancer patients in early stages with high AUC values (AUC > 84). Our method offers great potential for precision and early diagnosis of lung cancer in a non-invasive way based on urinary EVs towards clinical translations.

## Electronic supplementary material

Below is the link to the electronic supplementary material.

Supplementary Material 1

Supplementary Material 2

Supplementary Material 3

Supplementary Material 4

Supplementary Material 5

## Abbreviations

AUC: area under curve
DA: differential abundance
EVs: Extracellular vesicles
IDA: information-dependent acquisition
LC-MS/MS: liquid chromatography-tandem mass spectrometry
OPLS-DA: Orthogonal Partial Least Squares Discriminant Analysis
ROC: receiver operating characteristic
SDS-PAGE: sodium dodecyl sulfate-polyacrylamide gel electrophoresis
TME: tumor microenvironment.
