# Supplementary material for: Metabolomic investigation of urinary extracellular vesicles for early detection and screening of lung cancer
Source: J Nanobiotechnology. 2023 May 16;21:153. doi: 10.1186/s12951-023-01908-0 (PMC10186743; doi:10.1186/s12951-023-01908-0)
Supplement: Supplementary file 1 — Supplementary Material 1 [file 12951_2023_1908_MOESM1_ESM.docx]

**Metabolomic investigation of urinary extracellular vesicles for early detection and screening of lung cancer**

*Qinsi Yang^#a,b^, Jiaxin Luo^#a^, Hao Xu^#a^, Liu Huang^c^, Xinxi Zhu^d^, Henrrui Li^a^, Rui Yang^a^, Bo Peng^b^, Da Sue^e^, Qingfu Zhu^a^*, and Fei Liu^a^****

^a^National Engineering Research Center of Ophthalmology and Optometry, Eye Hospital, Wenzhou Medical University, Wenzhou, 325027, China

^b^Wenzhou Institute, University of Chinese Academy of Sciences, Wenzhou, Zhejiang 32500, China

^c^Department of Oncology, Tongji Hospital, Tongji Medical College, Huazhong University of Science and Technology, Wuhan, Hubei, 430030, China

^d^Key Laboratory of Heart and Lung, The First Affiliated Hospital of Wenzhou Medical University, Wenzhou, Zhejiang 325000, China.

^e^Institute of Life Sciences & Engineering Laboratory of Zhejiang Province for Pharmaceutical Development of Growth Factors, Wenzhou University, Wenzhou 325035, China;

*Correspondence to:

Qingfu Zhu, qingfu.zhu@wmu.edu.cn; Fei Liu, feiliu@wmu.edu.cn

***
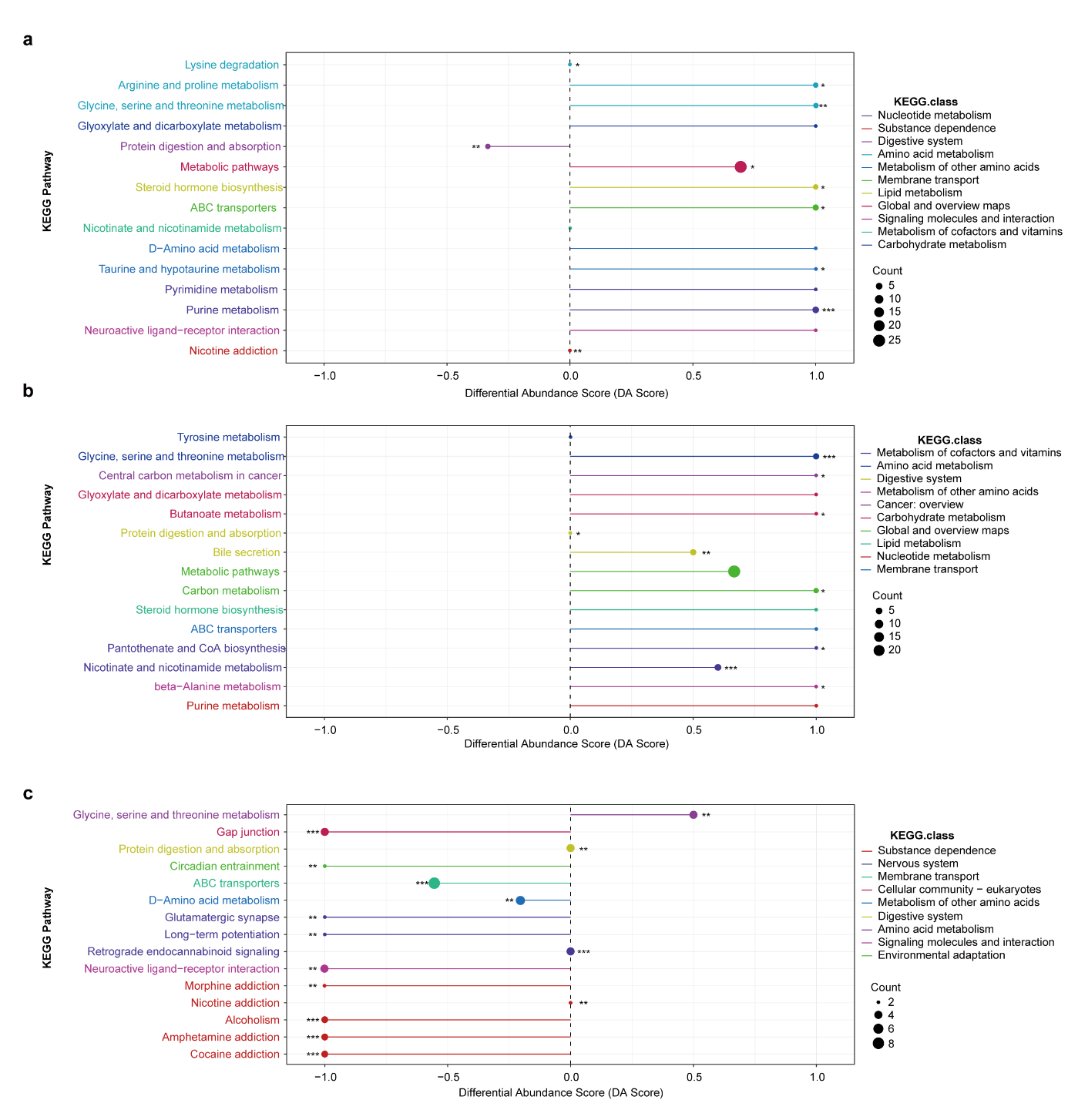
***

**Figure S1**. Compare the differential abundance (DA) scores of metabolic pathways between (a) lung cancer and healthy control, and (b) early lung cancer and healthy control. The DA score captures the average overall change for all metabolites in the pathway.

*
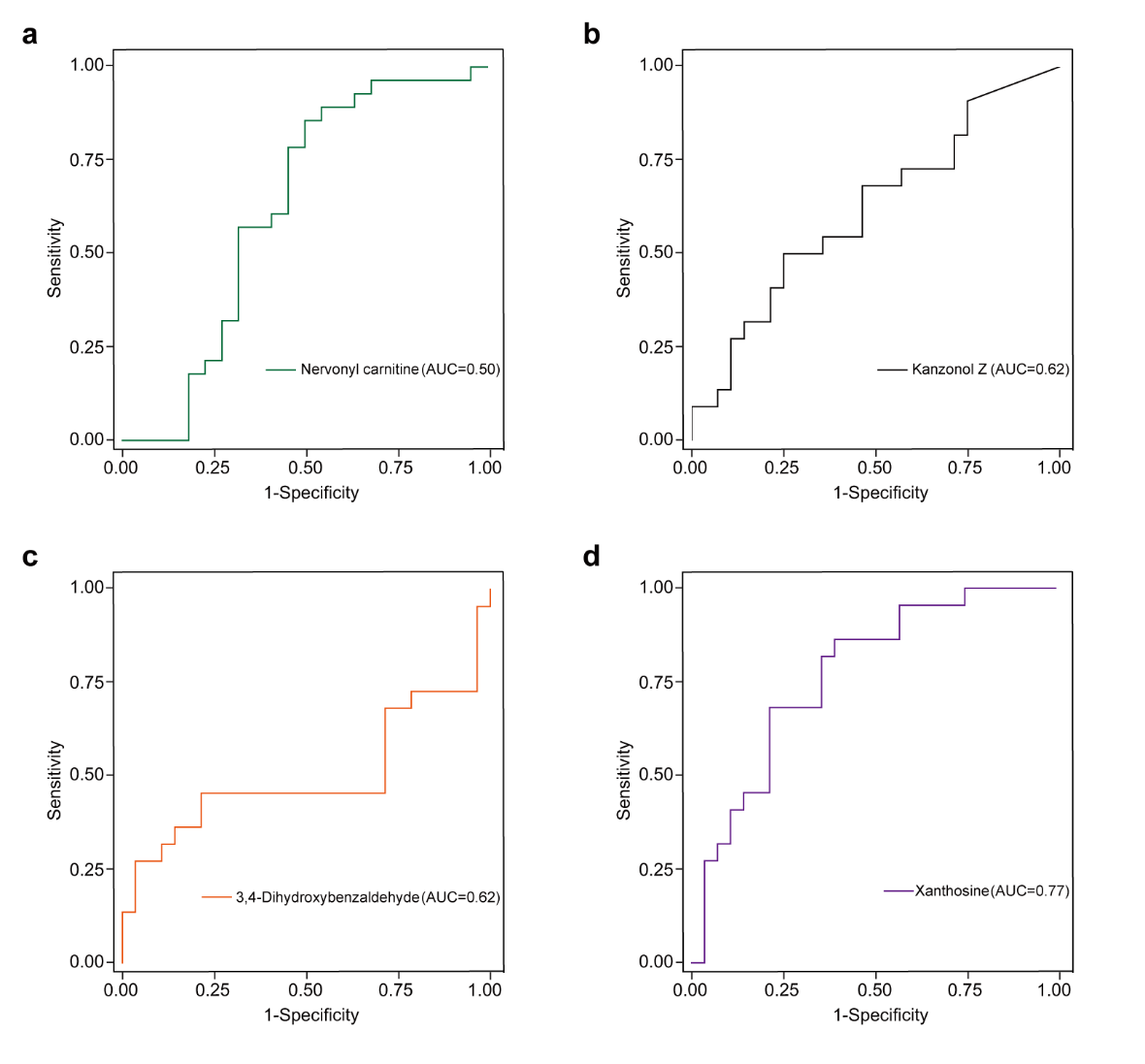
*

**Figure S2.** The receiver operating characteristics (ROC) curves and area under curve (AUC) values of 4 metabolites in the random forest model to distinguish early lung cancer patients from healthy controls.
